# Comprehensive Expression Profiling and Functional Network Analysis of p53-Regulated MicroRNAs in HepG2 Cells Treated with Doxorubicin

**DOI:** 10.1371/journal.pone.0149227

**Published:** 2016-02-17

**Authors:** Yalan Yang, Wenrong Liu, Ruofan Ding, Lili Xiong, Rongkun Dou, Yiming Zhang, Zhiyun Guo

**Affiliations:** School of Life Sciences and Bioengineering, Southwest Jiaotong University, Chengdu, Sichuan, P.R. China; Harbin Medical University, CHINA

## Abstract

Acting as a sequence-specific transcription factor, p53 tumor suppressor involves in a variety of biological processes after being activated by cellular stresses such as DNA damage. In recent years, microRNAs (miRNAs) have been confirmed to be regulated by p53 in several cancer types. However, it is still unclear how miRNAs orchestrate their regulation and function in p53 network after p53 activation in hepatocellular carcinoma (HCC). In this study, we used small RNA sequencing and systematic bioinformatic analysis to characterize the regulatory networks of differentially expressed miRNAs after the p53 activation in HepG2. Here, 33 miRNAs significantly regulated by p53 (12 up-regulated and 21 down-regulated) were detected between the doxorubicin-treated and untreated HepG2 cells in two biological replicates for small RNA sequencing and 8 miRNAs have been reported previously to be associated with HCC. Gene ontology (GO) and KEGG pathway enrichment analysis showed that 87.9% (29 out of 33) and 90.9% (30 out of 33) p53-regulated miRNAs were involved in p53-related biological processes and pathways with significantly low p-value, respectively. Remarkably, 18 out of 33 p53-regulated miRNAs were identified to contain p53 binding sites around their transcription start sites (TSSs). Finally, comprehensive p53-miRNA regulatory networks were constructed and analyzed. These observations provide a new insight into p53-miRNA co-regulatory network in the context of HCC.

## Introduction

The p53 tumor suppressor can be activated by different cellular stresses, such as DNA damage, oncogenic stresses and hypoxia [1]. As a transcription factor (TF), the p53 protein can activate or repress target gene expression and involves in various physiological processes such as apoptosis, cell cycle arrest and cell proliferation [2]. Previous study has demonstrated that p53 suppresses hepatocellular carcinoma (HCC) by activating its target genes such as p21 to inhibit cell proliferation and induce apoptosis in malignant cells [3]. In addition, the loss of p53 activity favors the development of liver tumor via dysregulating p53-mediated signaling pathways [4].

MicroRNAs (miRNAs) are a subset of endogenous non-coding RNAs (~22 nucleotides long) which play vital roles in regulating genes expression via targeting the specific sites in 3’ untranslated region (3’ UTR) of mRNAs [5]. The important roles of miRNAs in HCC development and progression have been verified by numerous experiments in recent years. For instance, miR-152 is related to the progression of HCC through deregulation of cell proliferation, motility and apoptosis by down-regulating Wnt-1, DNMT1, ERK1/2, AKT and TNFRS6B signaling pathways [6].

**Fig 1:**
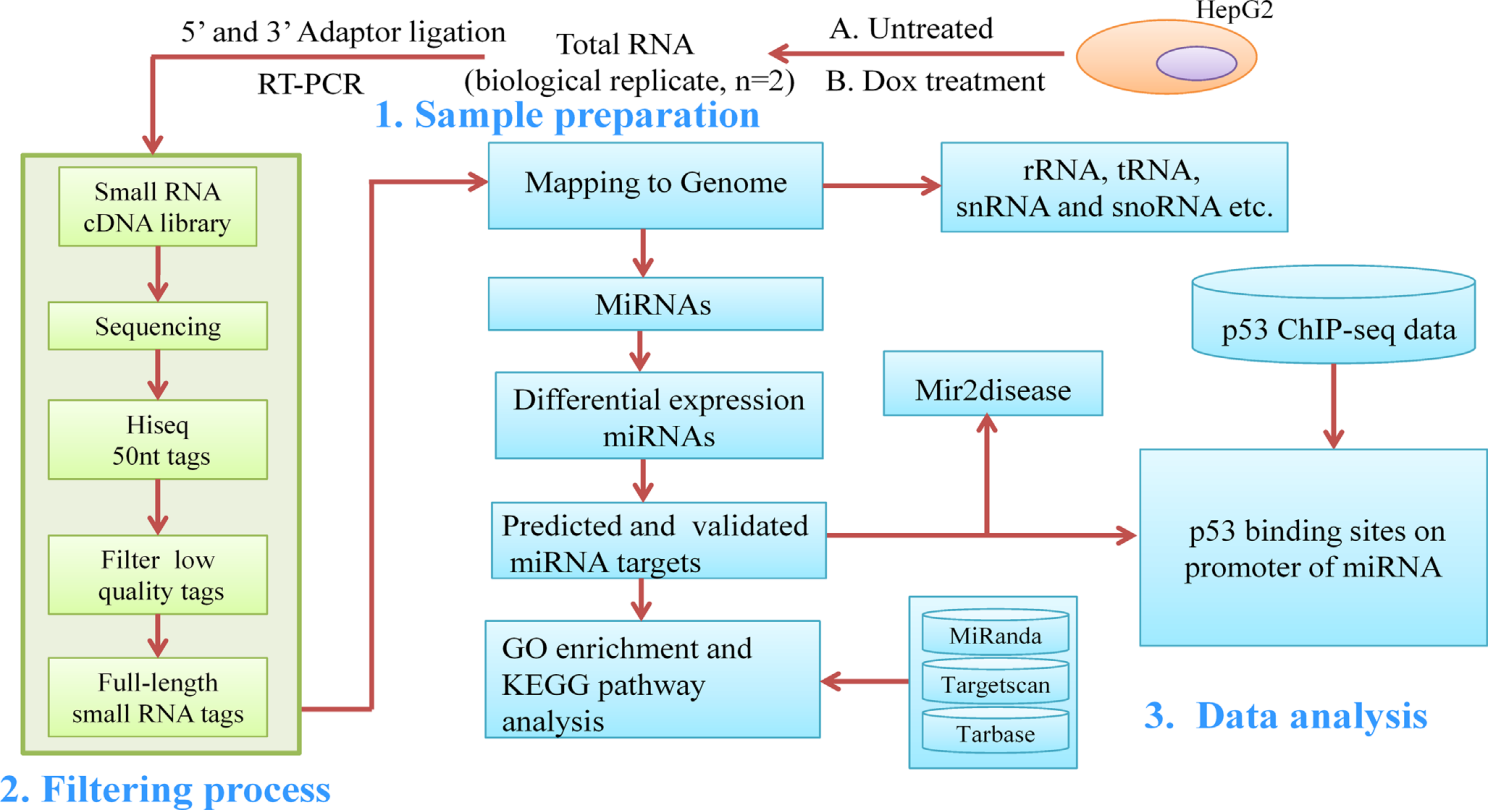
Flow chart of experimental design and systematic bioinformatic analysis in our research. The chart depicting the main 3 steps of experimental and computational pipeline employed in this study.

Previous studies reveal that miR-34 family can be regulated by p53 via binding to their promoters directly, which induce cell cycle arrest by suppressing the cell cycle-associated proteins in A549 and HCT116 cell lines [7]. Xi et al. analyzed the different expression profile of miRNA using MIRCHIP2 array. They found 11 up- and 43 down-regulated miRNAs in p53-expressing (HCT116 p53+/+) compared with p53-knockout (HCT116 p53−/−) human colorectal cancer cell lines [8]. In HCC, a study verified that miR-200 family members were directly modulated by p53 and involved in p53-regulated epithelial-mesenchymal transition by targeting ZEB1 and ZEB2 in C3A cells [9]. Another study has confirmed miR-509 was a direct transcriptional target of p53 and it regulated the cell cycle G1-S phase transition to inhibit the migration and proliferation of human hepatoma cells [10]. Although there have been reports that microRNA involved in p53 network, no studies have been undertaken to identify certain miRNAs directly regulated by p53 and construct p53-miRNA network in the context of HCC under DNA damage. Here we used small RNA sequencing (small RNA-seq) to explore the differential expression profiles of p53-dependent miRNAs and p53-miRNA regulatory networks in HCC cell line. The outline of experimental design and a range of systematic bioinformatic analysis are presented in Fig 1.

## Materials and Methods

### Cell culture and treatment of doxorubicin

HepG2 were purchased from ATCC and incubated with Dulbecco's Modified Eagle Medium (DMEM) (Gibco) with 10% fetal bovine serum (Hyclone), 100 IU/ml penicillin and 100 μg/ml streptomycin solution (Hyclone). Cell cultures were maintained at 37°C and at 5% CO_2_. 5.0×10^6^ HepG2 cells were plated in 60 mm dishes and cultured for 24h. For drug treatment, the growing cells were treated with 1μg/ml doxorubicin (Dox) (BeiJing HuaFeng). Treated cells were collected at different time points (0, 8, 16, 20 and 24h) for RNA and protein extraction.

### RNA Extraction and RT-qPCR

Total RNA was extracted from the cultured cells using Trizol Reagent (Invitrogen, USA) according to the manufacturer’s instructions. Determine the yield and purity of the RNA by measuring the absorbance of RNA samples at 260 and 280nm and verify the integrity of RNA samples using 1% agarose gel.

Total RNA was reverse transcribed to cDNA using oligo(dT) primers and M-MLV reverse transcriptase (Invitrogen, USA). GAPDH was considered as an endogenous control. The Bulge-Loop^TM^ miRNA qRT-PCR Primers were purchased from GuangZhou RiboBio.Co., Ltd, U6 small nuclear RNA was used as an endogenous control. Use FastStart Essential DNA Green Master (Roche) for real-time PCR according to the manufacturer’s protocol and all the samples were run in triplicate on lightcycler96 instrument (Roche Diagnostics, Switzerland). Cycling conditions were 95°C for 5min, followed by 45 cycles of 95°C for 10 sec, 60°C for 30sec and 72°C for 20sec.

### Western blotting

Cells were harvested after the treatment of Dox at different time points using 0.25% trypsin and collected by centrifugation after washing with phosphate buffer saline (PBS) three times. Total protein extracts were prepared in RIPA buffer supplemented with proteinase inhibitors. p53 antibody (DO-1) (dilution 1:500, sc-126, SantaCruz Biotechnology) and GAPDH antibody (3B3) (dilution 1:5000, M20006S, Abmart Inc.) were used for western blot analysis according to the manufacturer’s instructions. Goat Anti-Mouse IgG-HRP (dilution 1: 5000, M21001S, Abmart Inc.) were used as the secondary antibody. GAPDH was used as a protein loading control. The signals were visualized using the enhanced chemiluminescence (ECL) reagent (Thermo, USA).

### Small RNA library preparation and sequencing

Length of 18–30 nt small RNAs were isolated from 10μg total RNAs using 15% denaturing polyacrylamide gel electrophoresis. Small RNAs were ligated to a 5′ adaptor and a 3′ adaptor by T4-RNA ligase and reverse-transcribed to cDNA. HepG2 cells were treated with Dox for 0h and 24h twice, four libraries were obtained and marked as HepG2 0h-1, HepG2 24h-1, HepG2 0h-2, HepG2 24h-2. Agilent 2100 Bioanalyzer and ABI StepOnePlus Real-Time PCR System were applied to test the quality, purity and concentrations of the samples. Finally, cDNA libraries were sequenced using Illumina HiSeq 2000 platform (Illumina, San Diego, CA, USA) by following the manufacturer’s instructions. The raw data of HepG2 0h-1, HepG2 24h-1, HepG2 0h-2, HepG2 24h-2 were available at Gene Expression Omnibus (GEO) with the accession number: GSM1923400, GSM1923402, GSM1923401 and GSM1923403.

### Data analysis of small RNAs

The raw Illumina reads were subject to data cleaning procedures to remove contaminants from the 50nt sequencing tags, including low quality reads, reads with 5’ primer contaminants, reads without 3’ primer, the insert tags, reads without the insert tags and reads with poly A. The high-quality clean reads were mapped to human reference genome by SOAP [11] (allowing no more than one mismatch) for the analysis of their expression and distribution on the genome. SOAP is suitable for single-end reads and have advantage in accuracy than other short-read alignment tool such as Bowtie and BWA [12]. Blast was used to align the reads to miRBase database in order to identify the known miRNAs.

### Differential expression analysis of miRNAs

To identify the candidate differentially expressed miRNAs (DEMs) in HepG2, we compared the miRNA expression libraries between 0h and 24h. The relative expression of miRNAs in each library was analyzed by normalizing each miRNA count to the total count of clean reads. The normalized expression was used to calculate relative fold change (fold change = (normalized expression in treatment)**/**(normalized expression in control)). Significance of each differentially expressed miRNA was calculated using a statistical framework developed by Audic and Claverie [13]. P-values were adjusted for multi-testing procedures. The selection criteria for the most robustly differential expression of p53-related miRNAs were as followed: 1) the miRNAs expression level must be significantly induced (|log_2_ (fold change)| > 1) in two independent biological replicates, 2) reads counts > 10 in one sample at least, 3) miRNA expression must be significantly altered in at least one of these samples (p < 0.01).

### ChIP-Seq data analysis of p53

An attempt to characterize the p53-regulated miRNAs, available highly reliable p53 chromatin immunoprecipitation-sequencing (ChIP-seq) data from four studies were downloaded instead of computational recognition to explore potential p53 binding sites on the putative promoter region of miRNAs [14–17]. MiRNA TSSs were downloaded from miRStart database (http://mirstart.mbc.nctu.edu.tw/), a resource of human miRNA TSSs. For TSSs of miRNAs do not collected in miRStart, we defined their TSSs with the following steps: 1) obtain the genomic location of miRNAs from UCSC database, 2) TSSs of intragenic miRNAs were identified by transcription initiation of the host gene transcripts, 3) TSSs of the remaining miRNAs were determined by the genomic location of first nucleotide of each pre-miRNA. Next, 10 kb upstream and 1 kb downstream of TSS of each miRNA was defined as the putative promoter region based on previous studies [18, 19].

### MicroRNA target gene prediction, functional enrichment analysis and p53-miRNA network construction

MicroRNA target genes were predicted by Targetscan and MiRanda. Experimentally verified miRNA target genes were obtained from Tarbase database. Gene ontology (GO) enrichment analysis was performed by DAVID [20]. WEB-based gene set analysis toolkit (WebGestalt) [21] was used to perform the KEGG pathway analysis. The significance level was set at 0.05 (p-value < 0.05).

The p53-miRNA network was constructed by Cytoscape software [22]. Protein-protein interaction data files were downloaded from BioGRID (Version 3.2) [23], and interactions of miRNA target proteins were identified by Perl script.

## Results and Discussion

### Activation of p53 in HepG2 cells after treatment with DNA-damaging agents

**Fig 2:**
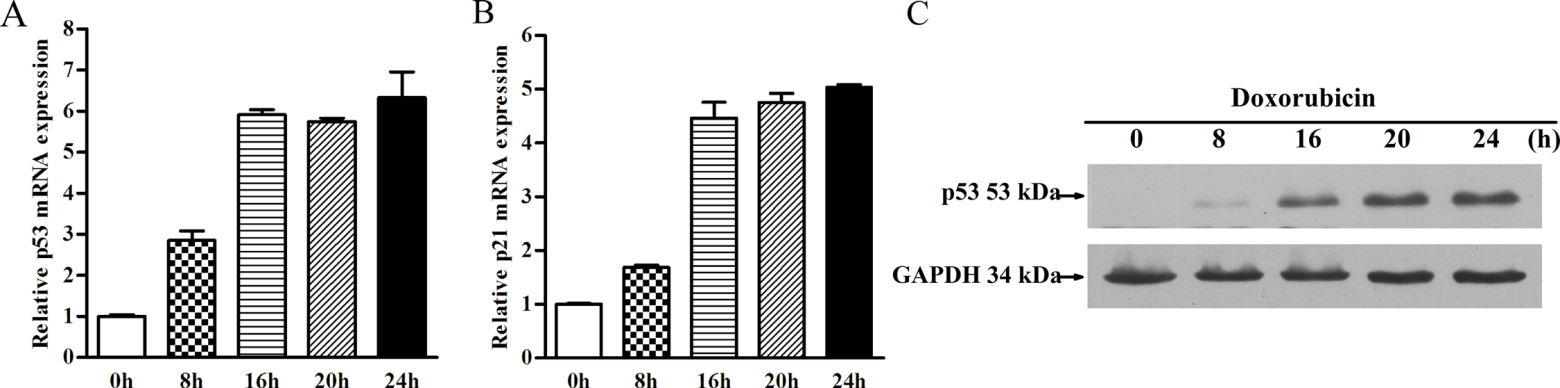
Doxorubicin induces p53 expression upon DNA damage. (A) HepG2 cells were treated with 1μg/ml Dox for 0, 8, 16, 20 and 24h. Expression fold changes of p53 were determined using real-time PCR. The expression of p53 increased significantly from 0 to 16h and stably expressed until 24h after Dox inducement; (B) p21, a well-known p53 downstream target gene, was chosen to validate the activation of p53 pathway after the Dox treatment; (C) Western blot analysis of p53 expression at 0, 8, 16, 20 and 24h after treatment with 1μg/ml Dox. The p53 expression at 24h after Dox treatment was significantly higher than in untreated HepG2 cells.

We used 1μg/ml Dox to induce the expression of p53 upon DNA damage at five time points (0, 8, 16, 20 and 24h) in HepG2 cells. The mRNA levels of p53 and p21 were detected by real-time RT-PCR. p21 is a well-known p53 target gene which was chosen to validate the activation of p53 pathway after Dox treatment. As expected, p53 and p21 were significantly up-regulated in mRNA level (6.33- and 5.04-fold change at 24h, respectively) in a time-dependent manner following Dox treatment (Fig 2A and 2B). Western blot analysis showed that p53 expression obviously increased in Dox-treated HepG2 cells compared with untreated cells and reached the maximum expression at 24h (Fig 2C). At 24 h post-Dox treatment, untreated and treated HepG2 cells were harvested for further small RNA sequencing.

### Length distribution, nucleotide bias and chromosome distribution characteristic of small RNAs

**Fig 3:**
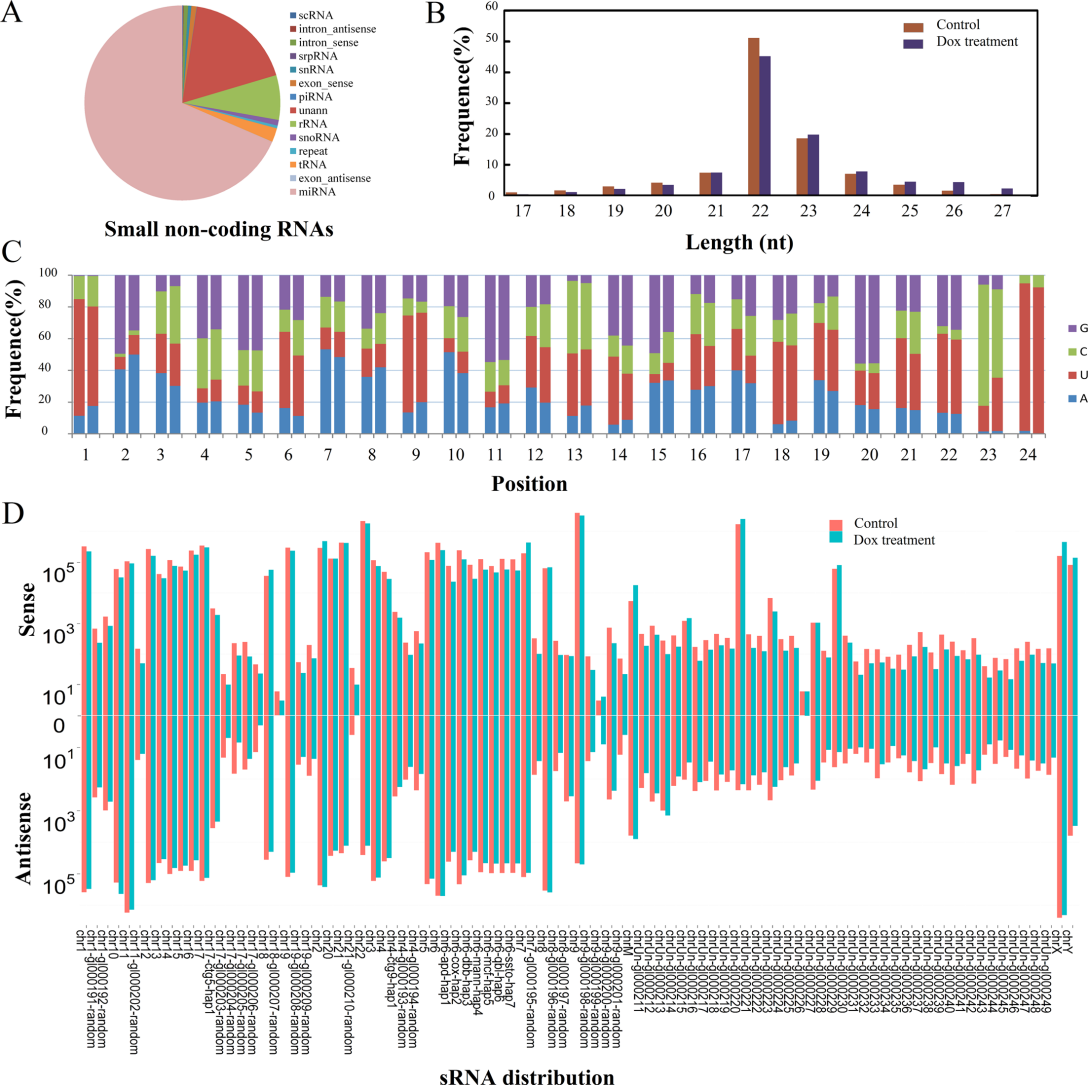
Characteristics of small RNAs in control and Dox-treated libraries. (A) Pie chart summarizing the annotation of small non-coding RNA species. MiRNAs occupy the highest proportion in total distribution of small RNA; (B) Length distribution of reads in control libraries and Dox-treated libraries. The horizontal coordinates represent reads lengths and the vertical coordinates represent percent of reads; (C) Nucleotide preference at each position of the known miRNAs in control libraries (left bar) and Dox-treated libraries (right bar). The horizontal coordinates are each position of sRNA reads and the vertical coordinates are percentages of AUCG at each base; (D) The distribution of sRNAs on different chromosomes in control libraries and Dox-treated libraries. The horizontal axes display distribution of chromosomes and the vertical axes display the count number of reads. Bars above axis: count number of reads with sense strand. Bars below axis: count number of reads with antisense strand.

Approximately 98% of raw reads were preserved after filtering the low quality reads in the four small RNA libraries (HepG2 0h-1, HepG2 24h-1, HepG2 0h-2 and HepG2 24h-2). After mapped clean reads to the human genome, the reads were categorized into different RNA species (Fig 3A). As expected, a total of 1211 known miRNAs were identified in this study and they occupied the highest proportion in total clean reads. To investigate the length distribution of reads in HepG2 cells, reads whose percent occupies beyond 1% of total reads and nucleotide length ranged from 17- to 27-nt were counted. We found that the majority of reads were ranged from 21- to 24-nt in length. And reads were significantly enriched in 22-nt length in both the control (51.41%) and Dox-treated libraries (45.14%) (Fig 3B), consistent with the vital majority of identified miRNAs which were composed of 22 nucleotides in metazoan [24].

The nucleotide bias analysis showed that uridine (U) occupied a major proportion (56.30%-73.50%) of first and ninth nucleotide of miRNAs, suggesting that first and ninth nucleotides were the 5’ and 3’ edges of mature miRNA seed region which were essential for the recognition of target mRNA and miRNA-mediated translational repression [25]. Guanine (G) was rarely present in the first base of miRNAs which were consistent with previous studies [25]. We also found that cytosine (C) and uridine (U) were more frequently observed at 23^th^ and 24^th^ position of mature miRNAs, respectively, suggesting these two positions may affect the identification of the cleavage at the trailing of mature miRNAs (Fig 3C). In addition, the nucleotide bias of miRNAs did not show significant differences between treatment and control groups. This result suggests that the base composition of these differentially expressed miRNAs do not exhibit sequence conservation feature under DNA damage compared with control. Finally, comparing sRNAs genome distribution of untreated cells with Dox-treated cells, we found that distributions of sRNAs appeared the similar feature in each chromosome, suggesting the consistency of genomic characterization of sRNA under the conditions of DNA damage (Fig 3D).

### Identification and analysis of differentially expressed miRNAs regulated by p53 in HepG2

**Fig 4:**
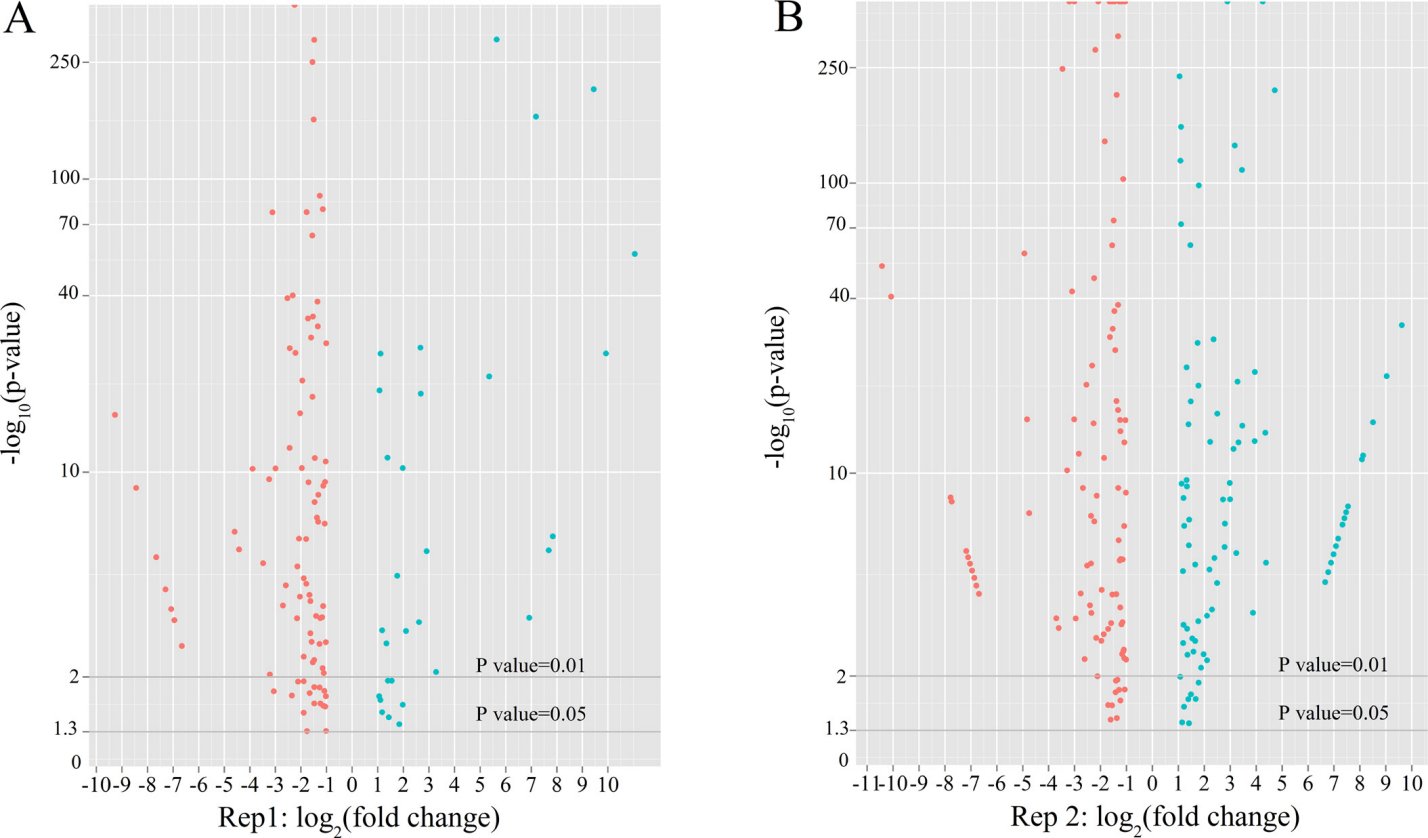
Differentially expressed miRNAs after Dox treatment. The scatter plots of DEMs in the two replicates: (A) HepG2 0h-1 vs 24h-1; (B) HepG2 0h-2 vs 24h-2. The horizontal coordinates are fold change (log_2_) and the vertical coordinates are the p value (-log_10_) (p-value < 0.05). Red plots: log_2_ (fold change) < -1, blue plots: log_2_ (fold change) > 1.

To identify the differentially expressed miRNAs (DEMs) associated with p53 more reliably in HepG2, two independent biological replicated samples with Dox-treated at 0h and 24h were sequenced on the Illumina HiSeq 2000 system. Finally, 127 and 215 DEMs were found with p-value < 0.05 in 2 independent replicates, respectively (Fig 4A and 4B).

**Fig 5:**
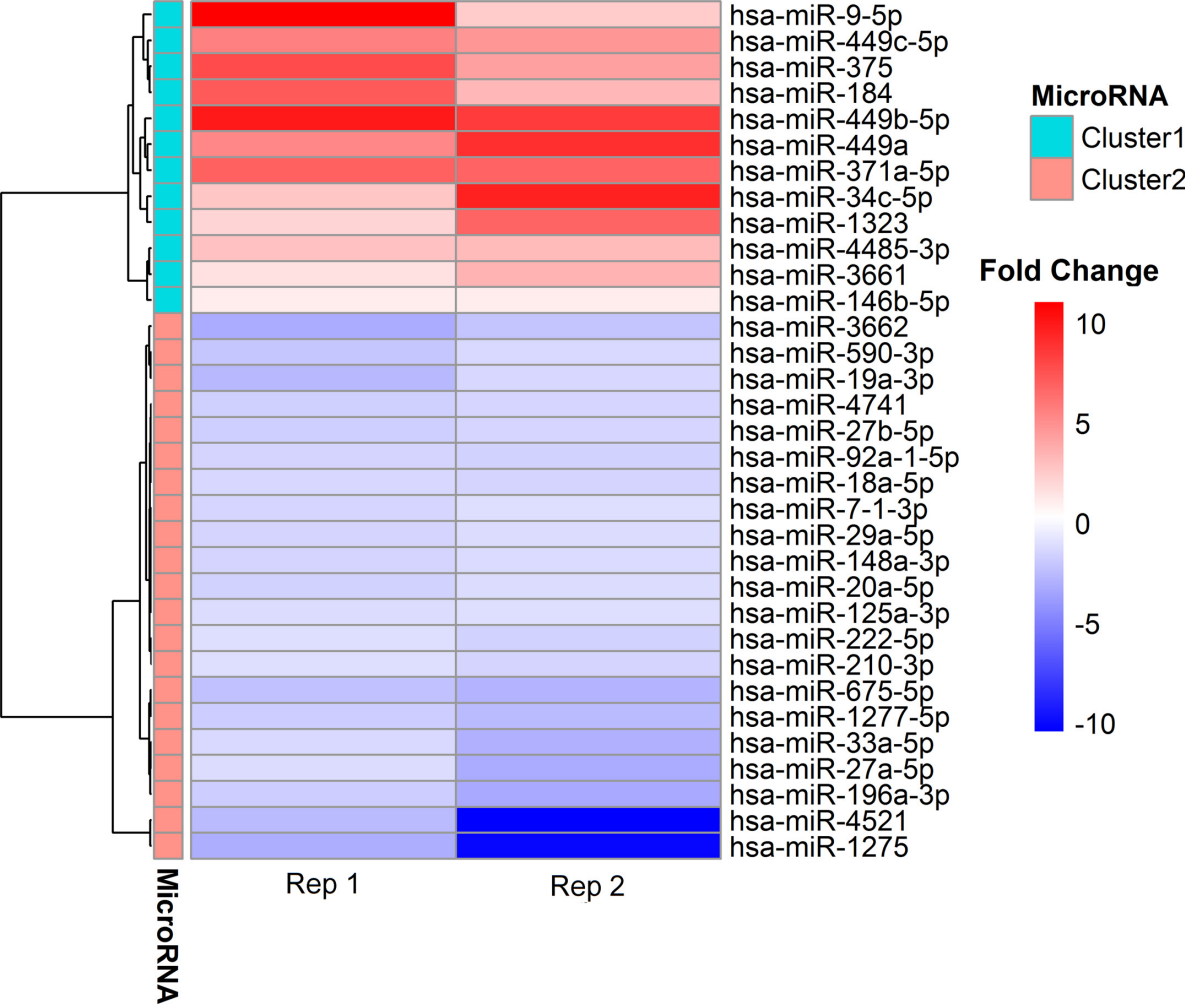
Heat map and cluster patterns of differentially expressed miRNAs between Dox-treated samples and control in HepG2. MiRNAs are clustered together with similar expression profile using hierarchical clustering.

To further characterize the p53-regulated miRNAs after the treatment of Dox, we focused on the most robustly-induced miRNAs using our selection criteria (see **Materials and Methods**). Finally, 33 miRNAs were identified and classified into two representative clusters based on the expression profile using hierarchical clustering (Fig 5). Cluster 1 contained 12 up-regulated miRNAs and cluster 2 contained 21 down-regulated miRNAs in two biological replicates (Table 1). In cluster 1, a verified p53 direct target hsa-miR-34c-5p [26] was highly induced after the treatment of Dox. Meanwhile, we discovered the highly up-regulated miR-449 cluster members: hsa-miR-449a, hsa-miR-449b-5p and hsa-449c-5p in our study. It has been confirmed that the expression of miR-449a and miR-449b-5p were 19- to 21-fold elevated after p53 activation in ovarian neoplasms [27]. In cluster 2, 4 out of 6 miR-17-92 cluster members (hsa-miR-18a, hsa-miR-19a, hsa-miR-20a, hsa-miR-92a-1) were all repressed after the activation of p53 protein. Previous findings indicated the similar results that miR-17-92 cluster were repressed due to the direct target of p53 via specific binding on the promoter region of miR-17-92 under hypoxic conditions [28]. Among the 33 DEMs, the differential expression profiles of several miRNAs have been identified in various cell lines induced by Dox. For example, the upregulation of miR-375 identified in our research was consistent with the results in multiple cancer cell lines (CAL27, SCC-25 and K562) under the Dox treatment [29, 30]. Moreover, a consistent pattern of expression of hsa-miR-1275, hsa-miR-27a-5p, hsa-miR-27b-5p and hsa-miR-449c-5p across Dox-treated cell lines (HepG2, MDA-MB-231, MDA-MB-468 and MCF-7) were found in both previous study and our research, suggesting an intimate correlation of these miRNAs regulated pattern with the Dox treatment [31]. These findings suggest that these miRNAs identified in our study have the DNA damage-specific regulation in the cancer cell lines.

**Table 1:** Differential expression of miRNAs derived from small RNA sequencing in HepG2 cell after activation of p53.

miRNA	Replicate 1			Replicate 2			Type	Host Gene
	log_2_(fold change)	p-value	Sig-lable	log_2_(fold change)	p-value	Sig-lable		
hsa-miR-675-5p	-2.26	0.00E+00	**	-2.84	2.09E-12	**	Intron/Exon	H19
hsa-miR-125a-3p	-1.16	7.18E-03	**	-1.08	1.67E-13	**	Intergenic	
hsa-miR-148a-3p	-1.46	6.74E-12	**	-1.21	0.00E+00	**	Intergenic	
hsa-miR-18a-5p	-1.37	1.01E-07	**	-1.47	6.01E-37	**	Intron	MIR17HG
hsa-miR-92a-1-5p	-1.51	2.20E-160	**	-1.55	1.00E-61	**	Intron	MIR17HG
hsa-miR-1277-5p	-1.8	1.22E-06	**	-2.54	6.31E-21	**	Intron	WDR44
hsa-miR-20a-5p	-1.56	6.44E-65	**	-1.12	4.98E-104	**	Intron	MIR17HG
hsa-miR-210-3p	-1.06	5.45E-10	**	-1.49	5.12E-75	**	Intergenic	
hsa-miR-222-5p	-1.03	1.91E-02	*	-1.53	3.62E-32	**	Intergenic	
hsa-miR-27a-5p	-1.14	2.53E-02	*	-3.2	0.00E+00	**	Intergenic	
hsa-miR-27b-5p	-1.73	3.66E-34	**	-1.38	7.43E-202	**	Intron	C9orf3
hsa-miR-29a-5p	-1.48	5.16E-03	**	-1.16	4.15E-03	**	Intergenic	
hsa-miR-19a-3p	-2.6	7.80E-05	**	-1.32	3.74E-321	**	Intron	MIR17HG
hsa-miR-33a-5p	-1.26	1.59E-88	**	-3.01	4.51E-16	**	Intron	SREBF2
hsa-miR-3662	-3.22	9.13E-03	**	-2.15	4.24E-09	**	Intron	HBS1L
hsa-miR-7-1-3p	-1.41	5.85E-04	**	-1.07	1.60E-02	*	Intron/Exon	HNRNPK
hsa-miR-4741	-1.66	1.72E-02	*	-1.43	2.86E-27	**	Intron	RBBP8
hsa-miR-590-3p	-2.04	1.74E-04	**	-1.3	1.32E-06	**	Intron	EIF4H
hsa-miR-196a-3p	-1.77	4.93E-02	*	-3.29	5.77E-11	**	Intergenic	
hsa-miR-4521	-2.53	5.56E-40	**	-10.43	1.77E-52	**	Intergenic	
hsa-miR-1275	-3.06	1.63E-02	*	-10.08	2.42E-41	**	Intergenic	
hsa-miR-146b-5p	1.07	9.65E-20	**	1.08	4.91E-120	**	Intergenic	
hsa-miR-449b-5p	9.92	4.06E-26	**	8.5	1.02E-15	**	Intron	CDC20B
hsa-miR-449c-5p	5.65	6.24E-300	**	4.72	1.57E-209	**	Intron	CDC20B
hsa-miR-371a-5p	6.92	6.48E-04	**	6.88	1.22E-05	**	Intergenic	
hsa-miR-184	7.19	6.39E-164	**	3.32	1.63E-13	**	Intergenic	
hsa-miR-34c-5p	2.68	2.96E-19	**	9.62	4.23E-33	**	Intergenic	
hsa-miR-3661	1.55	1.14E-02	*	3.46	1.16E-111	**	Intergenic	
hsa-miR-375	7.84	9.06E-07	**	4.25	0.00E+00	**	Intergenic	
hsa-miR-4485-3p	2.91	4.24E-06	**	3.17	1.62E-135	**	Intron	MTRNR2L8
hsa-miR-1323	2.11	1.34E-03	**	6.78	2.72E-05	**	Intergenic	
hsa-miR-449a	5.35	6.38E-22	**	9.03	2.52E-22	**	Intron	CDC20B
hsa-miR-9-5p	11.05	2.98E-56	**	2.5	8.76E-17	**	Intron	C1orf61

*: p<0.05

**: p<0.01.

**Fig 6:**
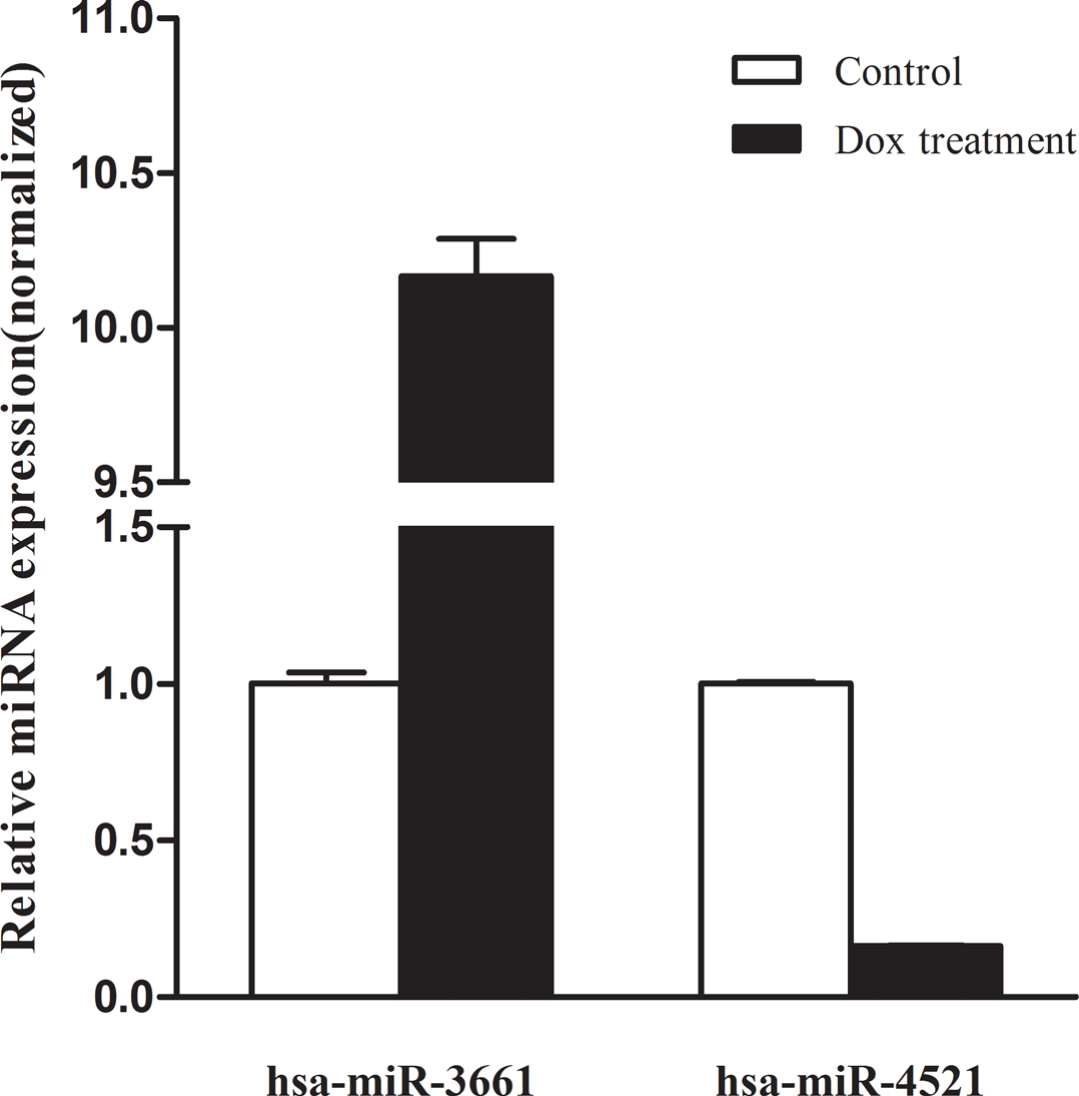
The relative expression of hsa-miR-3661 and hsa-miR-4521 after the p53 activation induced by Dox.

In this study, we identified several novel p53-regulated miRNAs in HCC including hsa-miR-1277, hsa-miR-146b, hsa-miR-3661, hsa-miR-3662, hsa-miR-371a, hsa-miR-4485 and hsa-miR-4521. Among them, hsa-miR-3662 was recently identified in lung cancer and was regarded as a potential early biomarker [32], indicating these new identified p53-regulated miRNAs may be critical regulators in HCC development and progression. Moreover, hsa-miR-3661 and hsa-miR-4521 were selected for qRT-PCR validation as they have never been studied in hepatocellular carcinoma. The results showed that hsa-miR-3661 was 10.17-fold increased and hsa-miR-4521 was down-regulated 6.25-fold which were consistent with sequencing results (Fig 6).

Furthermore, 33 selected miRNAs were injected into mir2disease, a database collecting human disease-associated miRNAs. Our findings showed that 18 out of 33 miRNAs were associated with various cancers, including pancreatic cancer, colorectal cancer, and breast cancer, etc (S1 Table). Remarkably, hsa-miR-125a, hsa-miR-148a, hsa-miR-19a, hsa-miR-27a, hsa-miR-18a, hsa-miR-20a, hsa-miR-222 and hsa-miR-9 were identified to relate to HCC. Among them, the relative expression of miR-27a in HCC tissues was significantly higher than that of their matched pericarcinomatous tissues. The similar results have been verified in hepatoma cell line HepG2 by comparison with normal human liver cell line HL-7702 [33]. These evidences indicated that miR-27a was shown highly specific expression in hepatocellular carcinoma than normal liver tissues or cells. Similarly, the comparison between human hepatocellular carcinoma samples and adjacent liver tissue showed that the expression of miR-18a, miR-19a and miR-20a were increased significantly in HCC [34]. These previous researches presented the crucial roles of these miRNAs in the development of malignant HCC. Moreover, the effects of these miRNAs in hepatocarcinogenesis have been elucidated and some of them appeared HCC tissue-specific manner. For instance, overexpressed miR-18a-5p was able to promote proliferation of HCC cell line HepG2 whereas repressed the proliferation of breast cancer cell line MCF-7 [35]. Some other studies reported that miR-27a was identified as an oncogenic miRNA in HCC which possessed the ability to promote proliferation, migration and invasion, but it played opposite roles as a tumor suppressor in acute leukemia by repressing anti-apoptotic gene 14-3-3θ [36, 37]. These studies suggest that we identified p53 regulated miRNAs play an essential role in the hepatocellular carcinoma development and progression.

### Identification of p53 binding sites in putative promoter region of DEMs

**Fig 7:**
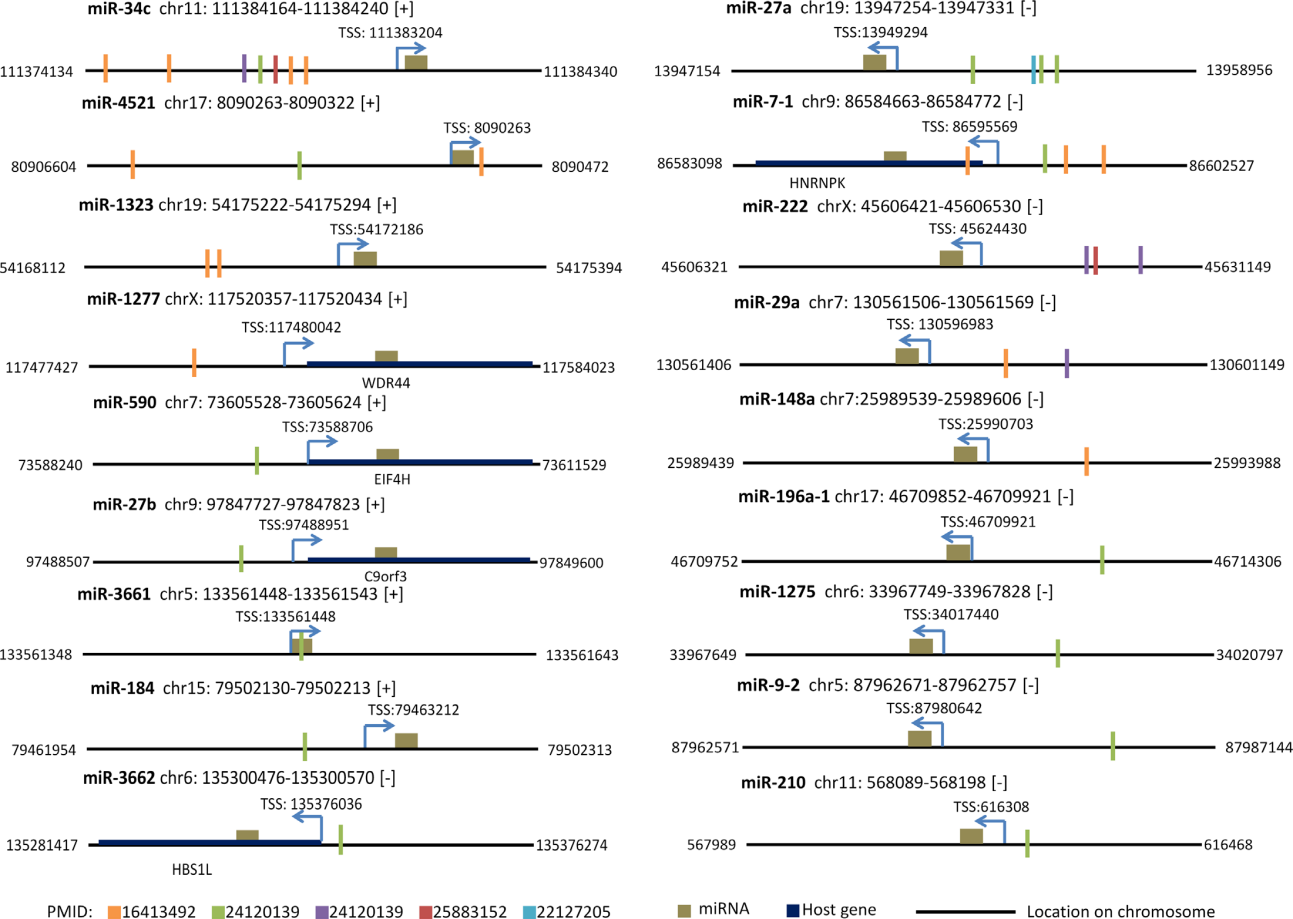
p53 binding sites around TSS of miRNAs. 18 miRNAs were identified to have potential p53 binding sites around TSSs. Orange, green, purple, red and blue bar represent location of p53 binding sites after analysis of p53-ChIP data from four studies PMID: 16413492, PMID: 24120139, PMID: 25883152 and PMID: 22127205, respectively. Dark goldenrod box represents miRNA, while thick dark blue line represents host gene. The thin black line represents the chromosome DNA.

After the analysis of p53 ChIP-seq data, eighteen (5 up-regulated and 13 down-regulated) out of 33 miRNAs have been identified the potential p53 binding sites around their TSSs and 7 miRNAs contain multiple predicted binding sites (Fig 7). Among these p53-regulated miRNAs, 8 miRNA transcripts located on plus strands whereas 10 located on minus strands. In summary, most (91.67%) of the p53-binding sites favored to locate on the upstream of TSSs of miRNAs except for miR-4521, miR-7-1 and miR-3661 (S2 Table). Particularly, 69.44% of p53 binding sites prefer to locate within the 5 kb upstream of TSSs. Previous TF-binding site enrichment analysis shown that TF-binding sites (TFBS) highly enriched in upstream of TSSs of miRNAs (69.6%) rather than in downstream regions (30.4%) which shows the similar distributions with our results [38]. Intriguingly, the p53 binding site of miR-3661 located within miRNA hairpin precursor instead of upstream or downstream of TSSs of miRNAs. Previous study has confirmed that the annotated TFBS can be found abundantly in pre-miRNA sequences after genome-wide analysis of conserved TFBS on human genome [39]. Taken together, p53-regulated miRNAs which contain p53 binding sites suggest that they are the potential positive or negative regulators of p53 which directly physically interacts with p53. However, excluding the reason of limited ChIP-seq dataset, other miRNAs that do not contain p53 binding sites are most probably as a member of p53 network mediated by p53 indirectly.

### Gene ontology (GO) and KEGG pathway enrichment analysis

**Fig 8:**
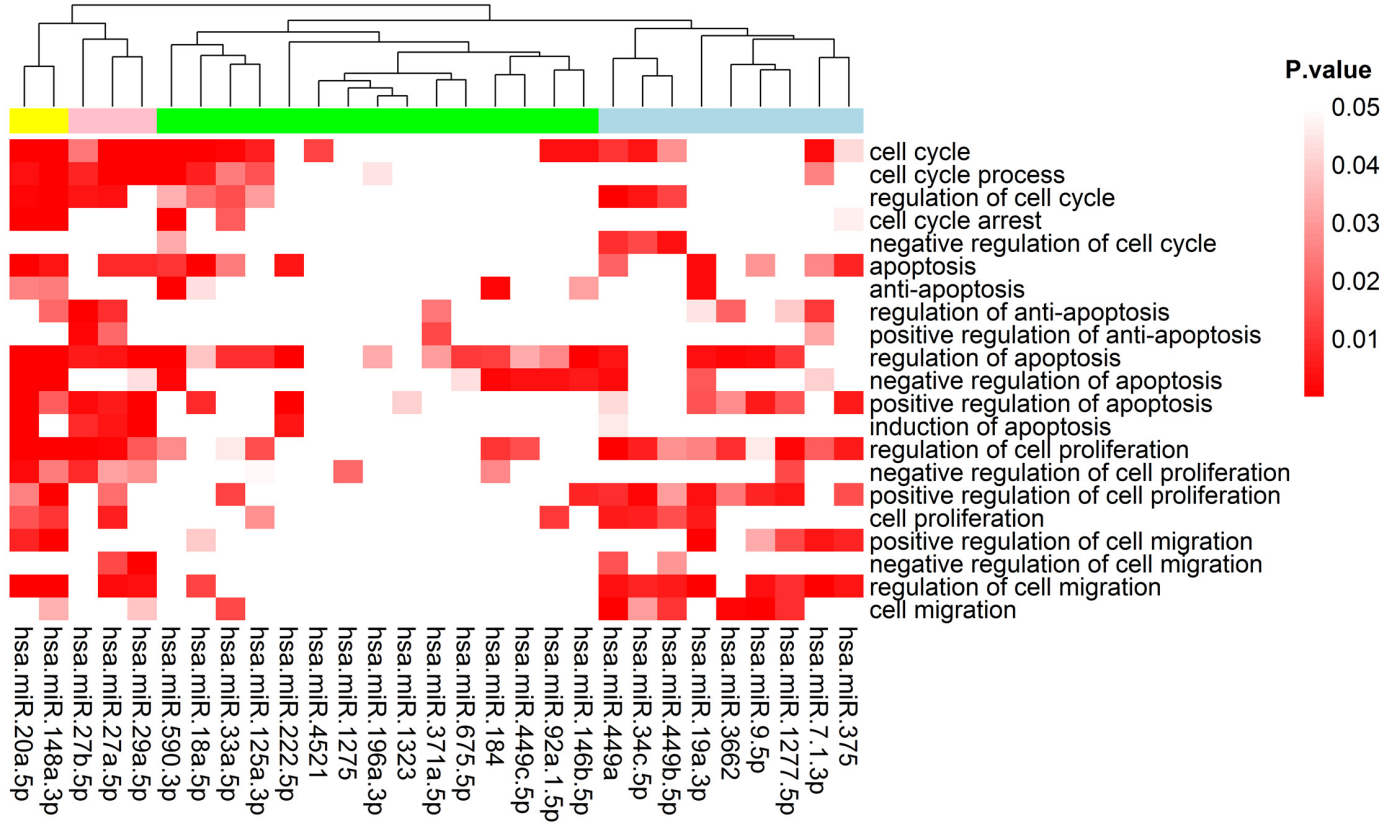
Heat map of gene ontology (GO) enrichment analysis of p53-miRNA target genes. 29 out of 33 miRNAs involved in biological processes associated with cancers (p-value < 0.05).

To gain insight into the functions of 33 p53-regulated DEMs in HepG2, we predicted miRNA target genes using widely accepted Targetscan and MiRanda algorithms. We choose Targetscan and MiRanda because they are the most popular miRNA target gene prediction tools and for the sake of getting a balance between reducing the false positive rate and reserving true positive results. To reduce false positives, target genes represented in both database were chosen for further analyses. Moreover, the experimentally verified miRNA target genes from Tarbase database were integrated into predicted miRNA target genes pool. Finally, we identified 4,752 predicted and 4,450 experimentally verified target genes, respectively. Next, the GO enrichment analysis was employed using the DAVID tools. Finally, 29 out of 33 miRNAs were discovered to participate in biological processes associated with tumorigenesis (Fig 8). Remarkably, hsa-miR-7-1-3p, hsa-miR-20a-5p, hsa-miR-27a-5p, hsa-miR-29a-5p, hsa-miR-148a-3p and hsa-miR-449a involved in most of the biological processes associated with cell cycle, apoptosis, proliferation and cell migration, indicating that these miRNAs may play vital roles in the p53-related biological processes. To determine target functions overall, we performed the GO enrichment analysis for the total of 7203 target genes of 33 miRNAs. As expected, we found most top ranked lists of enriched GO categories related to p53-mediated functions including: regulation of apoptosis (p-value = 1.80E-15), cell cycle (p-value = 7.30E-13) and regulation of cell proliferation (p-value = 1.70E-08), etc. (S3 Table**)**.

**Fig 9:**
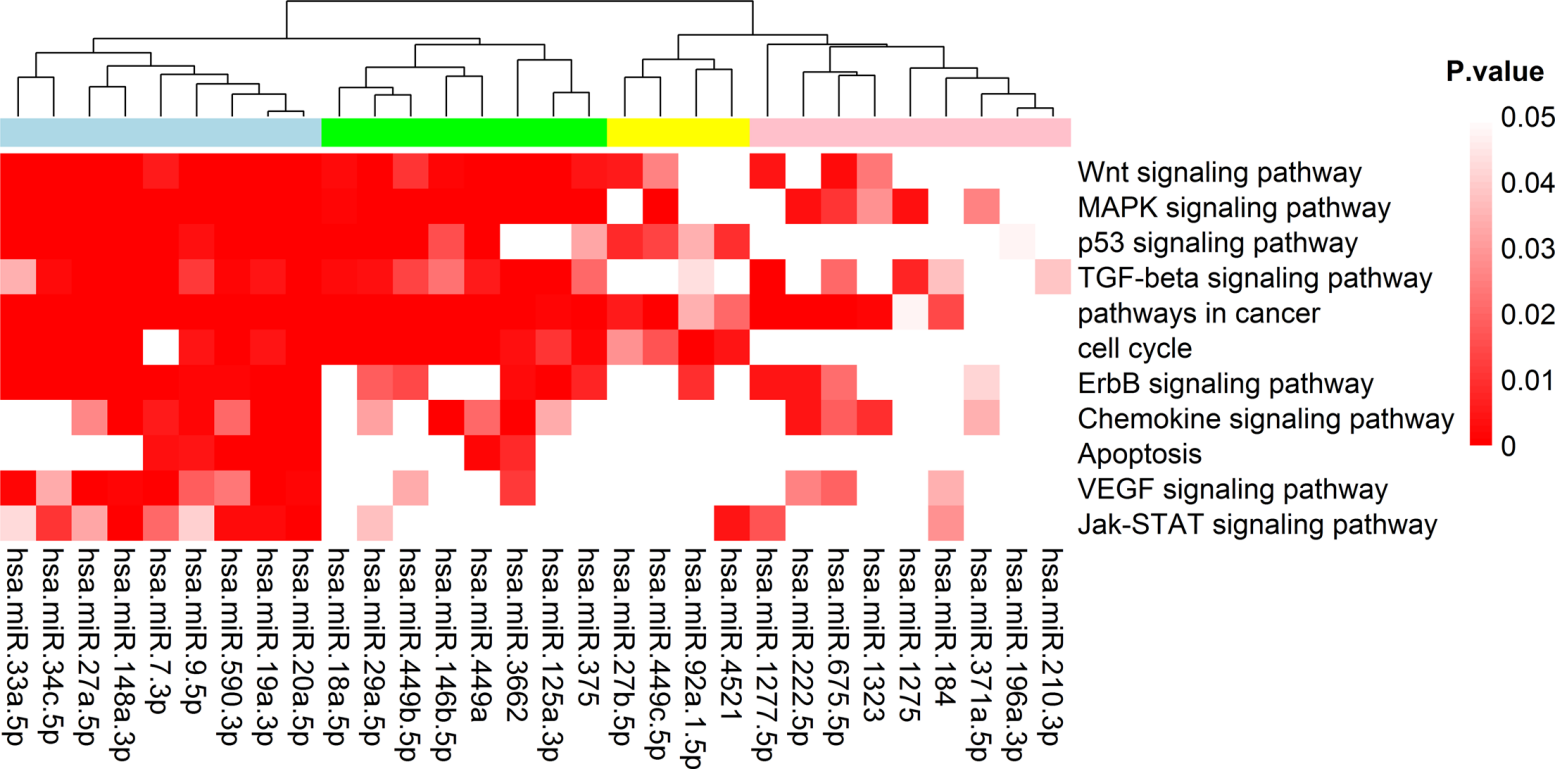
Heap map of KEGG Pathway enrichment analysis of p53-miRNA target genes. 30 out of 33 miRNAs participated in these pathways associated with tumorgenesis (p-value <0.05).

To identify the DEMs involved in cancer-related pathways, KEGG pathway analysis of their target genes were performed by WebGestalt. Our findings indicated that 30 out of 33 miRNAs were identified to participate in the regulation of cancer-related pathways (Fig 9). The results demonstrated 9 miRNAs (hsa-miR-33a-5p, hsa-miR-34c-5p, hsa-miR-27a-5p, hsa-miR-148a-3p, hsa-miR-7-3p, hsa-miR-9-5p, hsa-miR-590-3p, hsa-miR-19a-3p and hsa-miR-20a-5p) were significantly involved in most of the cancer-related signaling pathways with low p-value. In previous studies, various miRNAs have been experimentally verified to be involved in pathways described above. For instance, hsa-miR-33a-5p can inhibit cell growth by down-regulating the expression of β-catenin which affecting the Wnt pathway [40], hsa-miR-19a significantly decreased SOCS3 mRNA levels to enhance JAK-STAT signaling pathway [41] and hsa-miR-148a was found to be overexpressed in the Wnt signaling to suppress tumor activity or metastasis associated medulloblastomas [42], suggesting that those miRNAs play key roles in tumorigenesis. Next, we performed KEGG pathway analysis of all 7203 target genes of 33 miRNAs at the overall level. Notably, most of miRNAs target genes were involved in cancer-related signaling pathways with very low p-value ranged from 1.40E-73 to 6.08E-14 (S4 Table). The most well-represented pathway was pathways in cancer (p-value = 1.40E-73) in which p53 involved as a master regulator. In this pathway, cell apoptosis or proliferation can be induced by a series of miRNA target genes such as E2F3, c-myc (direct target of miR-590, miR-148a, miR-27a and miR-34c) and MDM2 (direct target of miR-590, miR-148a, miR-27a and miR-9-2). Furthermore, 10 out of 18 miRNAs with p53 binding sites were involved in this pathway, suggesting their central regulatory role in the p53 network.

**Fig 10:**
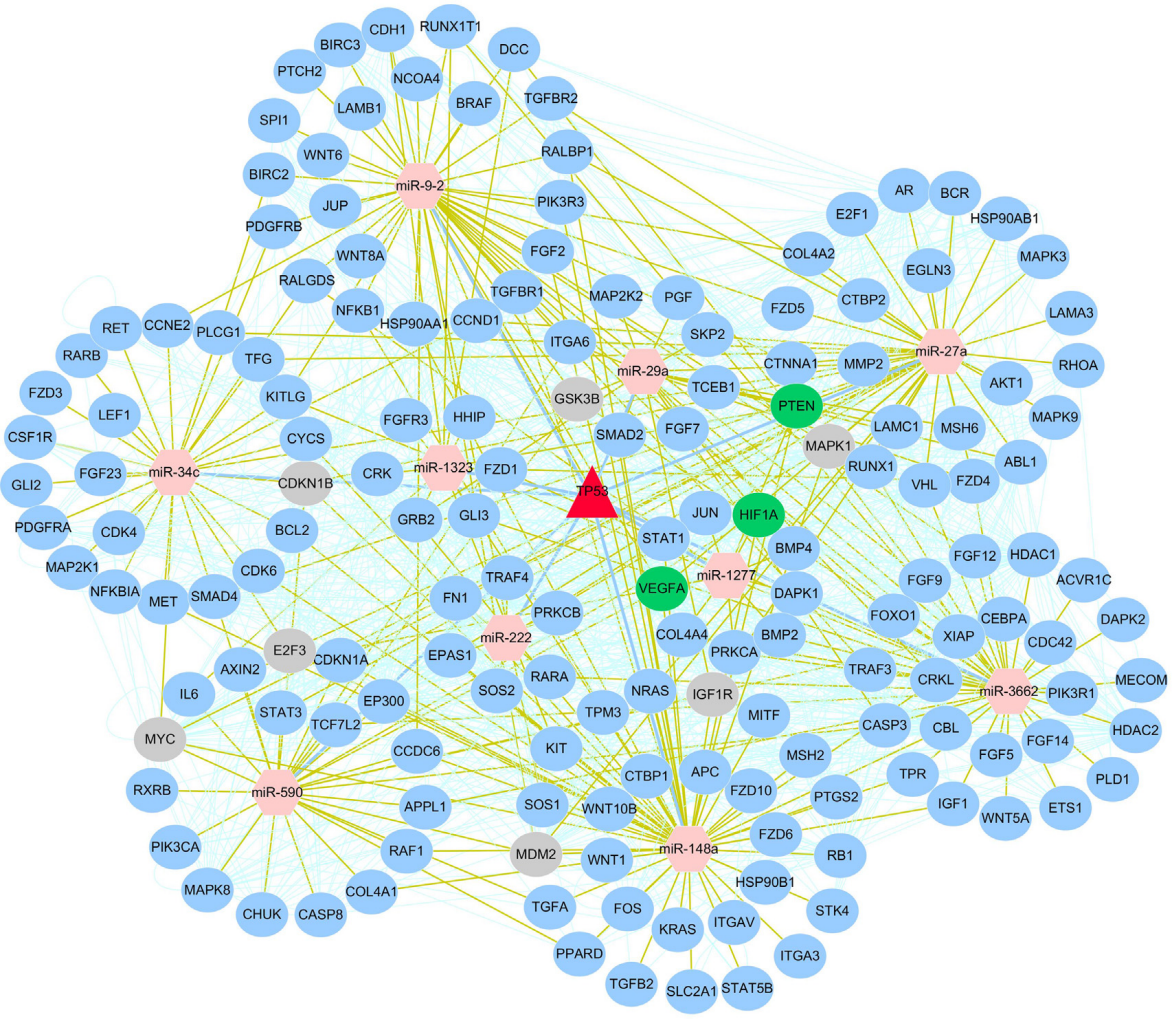
A curated p53-miRNA regulatory network was constructed using Cytoscape. Red triangle node represents p53 while pink hexagon nodes represent miRNAs with potential p53 binding sites on the putative promoter region of them. Blue circle nodes around the corresponding miRNA represent mRNAs targeted by 1~3 miRNAs, grey and green circle nodes represent mRNAs targeted by four and five miRNAs, respectively. p53-miRNAs: blue solid line, miRNAs-target genes: yellow solid line, protein-protein interaction: light green solid line.

For this purpose, we addressed the co-regulatory network of these 10 p53-miRNAs through employing protein–protein interactions (PPIs) from BioGRID database (Fig 10). We explored 64 miRNA-mediated feed-forward loops (FFLs), where p53 regulates a miRNA and both of them regulate a target together, such as p53-miR-34c-BCL2, p53-miR-222-MAPK1 and p53-miR-29a-PTEN (S5 Table). BCL2 was a known target of miR-34c that also co-regulated by p53 in oral metastatic tissues to facilitate tumor progression and metastasis [43]. Another study verified that p53 direct target miR-29a [44] was responsible for cell migration through its target gene PTEN [45]. In summary, we provide comprehensive and systematic function analysis of DEMs and their target genes, which provide new insights into the mechanism underlying the function of p53-regulated miRNAs and target genes in tumor suppression.

## Supporting Information

S1 Table Diseases associate with miRNAs in cancers. (DOCX)

S2 Table Potential p53 binding sites around their TSSs after analyzing p53 ChIP-seq data. (DOCX)

S3 Table Significant GO terms of total miRNA targets related to cancer. (DOCX)

S4 Table Significantly enriched KEGG pathways targeted by miRNAs. (DOCX)

S5 Table miRNA-mediated feed-forward loops in pathways in cancer. (XLSX)
