# Supplementary material for: Comprehensive Expression Profiling and Functional Network Analysis of p53-Regulated MicroRNAs in HepG2 Cells Treated with Doxorubicin
Source: PLoS One. 2016 Feb 17;11(2):e0149227. doi: 10.1371/journal.pone.0149227 (PMC4757586; doi:10.1371/journal.pone.0149227)
Supplement: S1 Table — (DOCX) [file pone.0149227.s001.docx]

**Table S1.** Diseases associated with miRNAs in cancers. Here, 8 miRNAs were found to have association with the development of HCC.

| **miRNA** | | **Disease** | |
| --- | --- | --- | --- |
| **hsa-miR-125a** | | breast cancer, acute lymphoblastic leukemia (ALL), colorectal cancer, epithelial ovarian cancer (EOC), **hepatocellular carcinoma (HCC)**, lung cancer, neuroblastoma (NB), prostate cancer, serous ovarian cancer, ovarian cancer (OC), medulloblastoma, acute myeloid leukemia (AML), pancreatic cancer | |
| **hsa-miR-148a** | | **hepatocellular carcinoma (HCC)**, breast cancer, oral squamous cell carcinoma (OSCC), pancreatic cancer, prostate cancer, pancreatic ductal adenocarcinoma (PDAC), head and neck squamous cell carcinoma (HNSCC) | |
| **hsa-miR-19a** | | **hepatocellular carcinoma (HCC)**, glioma, head and neck squamous cell carcinoma (HNSCC), colorectal cancer, anaplastic thyroid carcinoma (ATC), lung cancer, B-cell chronic lymphocytic leukemia, malignant lymphoma, medulloblastoma, multiple myeloma (MM) | |
| **hsa-miR-20a** | | glioma, chronic myeloid leukemia (CML), breast cancer, primary biliary cirrhosis (PBC), kidney cancer, lung cancer, Hodgkin's lymphoma, colorectal cancer, **hepatocellular carcinoma (HCC)**, medulloblastoma, colorectal cancer, B-cell lymphoma, malignant lymphoma, pancreatic cancer, prostate cancer, serous ovarian cancer | |
| **hsa-miR-210** | | acute myeloid leukemia (AML), glioma, head and neck cancer, acute promyelocytic leukemia (APL), glioblastoma multiforme (GBM), breast cancer, diffuse large B-cell lymphoma (DLBCL), acute lymphoblastic leukemia (ALL), follicular lymphoma (FL), head and neck squamous cell carcinoma (HNSCC), pancreatic cancer, lung cancer, kidney cancer, malignant melanoma, pancreatic ductal adenocarcinoma (PDAC), prostate cancer | |
| **hsa-miR-222** | | prostate cancer, tongue squamous cell carcinoma, acute lymphoblastic leukemia (ALL), **hepatocellular carcinoma (HCC)**, papillary thyroid carcinoma (PTC), glioblastoma, glioma, gastric cancer (stomach cancer), bladder cancer, malignant melanoma, breast cancer, pancreatic cancer, pancreatic ductal adenocarcinoma (PDAC), non-small cell lung cancer (NSCLC), acute myeloid leukemia (AML) | |
| **hsa-miR-27a** | | **hepatocellular carcinoma (HCC)**, acute lymphoblastic leukemia (ALL), colorectal cancer, malignant melanoma, oral squamous cell carcinoma (OSCC), prostate cancer, breast cancer, kidney cancer, acute myeloid leukemia (AML), gastric cancer (stomach cancer), autism spectrum disorder (ASD), cardiac hypertrophy, serous ovarian cancer, uterine leiomyoma (ULM) | |
| **hsa-miR-27b** | | acute lymphoblastic leukemia (ALL), colorectal cancer, lung cancer, oral squamous cell carcinoma (OSCC), prostate cancer, acute myeloid leukemia (AML), cardiac hypertrophy | |
| **hsa-miR-29a** | | neuroblastoma (NB), oral squamous cell carcinoma (OSCC), lung cancer, , prostate cancer, serous ovarian cancer, kidney cancer, colorectal cancer, ovarian cancer (OC) | |
| **hsa-miR-7-1** | | pituitary adenoma | |
| **hsa-miR-146b** | | breast cancer, glioma, lung cancer, ovarian cancer (OC), papillary thyroid carcinoma (PTC), head and neck squamous cell carcinoma (HNSCC) | |
| **hsa-miR-184** | | acute myeloid leukemia (AML), prostate cancer, glioma, epithelial ovarian cancer (EOC), malignant melanoma, oral squamous cell carcinoma (OSCC), neuroblastoma (NB), prostate cancer, adrenocortical carcinoma | |
| **hsa-miR-34c** | | pancreatic cancer, non-small cell lung cancer (NSCLC), colorectal cancer, ovarian cancer (OC), acute myeloid leukemia (AML), malignant melanoma, nasopharyngeal carcinoma (NPC), oral squamous cell carcinoma (OSCC), | |
| **hsa-miR-375** | | esophageal cancer, pancreatic cancer, pancreatic ductal adenocarcinoma (PDAC), prostate cancer, head and neck squamous cell carcinoma (HNSCC) | |
| **hsa-miR-449a** | | prostate cancer | |
| **hsa-miR-9** | | glioma, recurrent ovarian cancer, malignant mesothelioma (MM), ovarian cancer (OC), epithelial ovarian cancer (EOC), lung cancer, malignant melanoma, neuroblastoma (NB), non-small cell lung cancer (NSCLC), medulloblastoma, Hodgkin's lymphoma, **hepatocellular carcinoma (HCC)**, follicular lymphoma (FL) | |
| **hsa-miR-18a** | Hodgkin's lymphoma, breast cancer, medulloblastoma, colorectal cancer, **hepatocellular carcinoma (HCC)**, anaplastic thyroid carcinoma (ATC), lung cancer, pancreatic ductal adenocarcinoma (PDAC) | |  |
| **hsa-miR-196a** | pancreatic ductal adenocarcinoma (PDAC), Hodgkin's lymphoma, breast cancer, colorectal cancer, esophageal cancer | |  |
