# Supplementary material for: Comprehensive Expression Profiling and Functional Network Analysis of p53-Regulated MicroRNAs in HepG2 Cells Treated with Doxorubicin
Source: PLoS One. 2016 Feb 17;11(2):e0149227. doi: 10.1371/journal.pone.0149227 (PMC4757586; doi:10.1371/journal.pone.0149227)
Supplement: S2 Table — (DOCX) [file pone.0149227.s002.docx]

**Table S2.** 18 miRNAs obtain the potential p53 binding sites around their TSS after analyzing p53 ChIP-seq data from four studies.

| **miRNA name** | **Type** | **Strand** | **TSS of miRNA** | **p53 binding site** | **Distance** |
| --- | --- | --- | --- | --- | --- |
| hsa-miR-34c | Intergenic | + | Chr11: 111383204 | chr11: 111374234-111374521 [11] | -8970 |
|  |  |  |  | chr11: 111378777-111379435[11] | -4427 |
|  |  |  |  | chr11: 111381192-111381450[11] | -2012 |
|  |  |  |  | chr11:111381234-111382084[11] | -1970 |
|  |  |  |  | chr11:111380831-111381408[12] | -2373 |
|  |  |  |  | chr11:111380663-111381668[12] | -2541 |
|  |  |  |  | chr11:111380907-111381951[13] | -2297 |
| hsa-miR-27a | Intergenic | - | Chr19:13949294 | chr19:13951866-13952665[12] | 3371 |
|  |  |  |  | chr19:13953722-13958856[12] | 9562 |
|  |  |  |  | chr19:13953305-13953765[12] | 4471 |
|  |  |  |  | chr19:13953222-13953722[14] | 4428 |
| hsa-miR-7-1 | Intron | - | Chr9:86595569 | chr9:86595399-86596057[11] | 488 |
|  |  |  |  | chr9:86601499-86602427[11] | 6858 |
|  |  |  |  | chr9:86594648-86595021[11] | -548 |
|  |  |  |  | chr9:86595573-86595911[12] | 342 |
| hsa-miR-222 | Intergenic | - | ChrX:45624430 | chrX:45628991-45629681[12] | 5251 |
|  |  |  |  | chrX:45630481-45631049[12] | 6619 |
|  |  |  |  | chrX:45629203-45629639[13] | 5209 |
| hsa-miR-4521 | Intergenic | + | Chr17:8090263 | chr17:8083822-8084442[11] | -6441 |
|  |  |  |  | chr17:8090372-8090584[11] | 109 |
|  |  |  |  | chr17:8089298-8089974[12] | -965 |
| hsa-miR-29a | Intergenic | - | Chr7: 130596983 | chr7:130600614-130601049[11] | 4066 |
|  |  |  |  | chr7:130597851-130598552[12] | 1569 |
| hsa-miR-1323 | Intergenic | + | Chr19:54172186 | chr19:54168212-54168864[11] | -3974 |
|  |  |  |  | chr19:54168833-54172186[11] | -3353 |
| hsa-miR-148a | Intergenic | - | Chr7: 25990703 | chr7:25993219-25993888[11] | 3185 |
| hsa-miR-1277 | Intron | + | ChrX:117480042 | chrX:117477437-117478068[11] | -2605 |
| hsa-miR-196a-1 | Intergenic | - | Chr17: 46709921 | chr17:46713866-46714206[12] | 4285 |
| hsa-miR-590 | Intron | + | Chr7: 73588706 | chr7:73588340-73588979[12] | -366 |
| hsa-miR-1275 | Intergenic | - | Chr6:34017440 | chr6:34020352-34020697[12] | 3257 |
| hsa-miR-9-2 | Intergenic | - | Chr5:87980642 | chr5:87986771-87987044[12] | 6402 |
| hsa-miR-27b | Intron | + | Chr9: 97488951 | chr9:97488607-97489196[12] | -344 |
| hsa-miR-210 | Intergenic | - | Chr11:616308 | chr11:615584-616368[12] | 60 |
| hsa-miR-3661 | Intergenic | + | Chr5:133561448 | chr5:133561527-133561937[12] | 79 |
| hsa-miR-3662 | Intron | - | Chr6:135376036 | chr6:135375719-135376174[12] | 138 |
| hsa-miR-184 | Intergenic | + | Chr15: 79463212 | chr15:79462054-79462673[11] | -1158 |

**Distance**: the distance between TSS and p53 binding site. For strand (+): Distance<0 stands for p53 binding sites located in upstream of TSS, Distance>0 stands for p53 binding sites located in downstream of TSS. For strand (-): Distance<0 stands for p53 binding sites located in downstream of TSS, Distance>0 stands for p53 binding sites located in upstream of TSS.
