# Supplementary material for: Comprehensive Expression Profiling and Functional Network Analysis of p53-Regulated MicroRNAs in HepG2 Cells Treated with Doxorubicin
Source: PLoS One. 2016 Feb 17;11(2):e0149227. doi: 10.1371/journal.pone.0149227 (PMC4757586; doi:10.1371/journal.pone.0149227)
Supplement: S3 Table — (DOCX) [file pone.0149227.s003.docx]

**Table S3.** Significant GO terms of total miRNA targets related to cancer.

| **GO ID** | **Name** | **Gene Number** | **%** | **P-Value** |
| --- | --- | --- | --- | --- |
| GO:0042981 | regulation of apoptosis | 442 | 6 | 1.80E-15 |
| GO:0044838 | cell cycle | 418 | 5.7 | 7.30E-13 |
| GO:0042127 | regulation of cell proliferation | 402 | 5.5 | 1.70E-08 |
| GO:0006915 | apoptosis | 320 | 4.4 | 3.20E-09 |
| GO:0022402 | cell cycle process | 301 | 4.1 | 7.30E-09 |
| GO:0008283 | cell proliferation | 243 | 3.3 | 1.20E-09 |
| GO:0043065 | positive regulation of apoptosis | 231 | 3.1 | 2.00E-07 |
| GO:0008284 | positive regulation of cell proliferation | 212 | 2.9 | 4.30E-05 |
| GO:0043066 | negative regulation of apoptosis | 204 | 2.8 | 7.10E-10 |
| GO:0051726 | regulation of cell cycle | 186 | 2.5 | 5.60E-08 |
| GO:0008285 | negative regulation of cell proliferation | 185 | 2.5 | 1.30E-04 |
| GO:0016477 | cell migration | 168 | 2.3 | 8.70E-11 |
| GO:0006915 | induction of apoptosis | 166 | 2.3 | 1.30E-04 |
| GO:0043066 | anti-apoptosis | 116 | 1.6 | 1.90E-05 |
| GO:0030334 | regulation of cell migration | 106 | 1.4 | 3.90E-08 |
| GO:0007050 | cell cycle arrest | 66 | 0.9 | 6.90E-06 |
| GO:0030335 | positive regulation of cell migration | 60 | 0.8 | 1.80E-06 |
| GO:0045786 | negative regulation of cell cycle | 47 | 0.6 | 3.70E-03 |
| GO:0045767 | regulation of anti-apoptosis | 30 | 0.4 | 1.30E-05 |
| GO:0045768 | positive regulation of anti-apoptosis | 25 | 0.3 | 1.80E-05 |
