# Supplementary material for: Comprehensive Expression Profiling and Functional Network Analysis of p53-Regulated MicroRNAs in HepG2 Cells Treated with Doxorubicin
Source: PLoS One. 2016 Feb 17;11(2):e0149227. doi: 10.1371/journal.pone.0149227 (PMC4757586; doi:10.1371/journal.pone.0149227)
Supplement: S4 Table — (DOCX) [file pone.0149227.s004.docx]

**Table S4.** Significantly enriched KEGG pathways targeted by miRNAs.

| **KEGG ID** | **Pathway** | **Gene Number** | **P-value** |
| --- | --- | --- | --- |
| map05200 | Pathways in cancer | 206 | 1.40E-73 |
| map04010 | MAPK signaling pathway | 159 | 9.86E-52 |
| map04310 | Wnt signaling pathway | 94 | 1.49E-33 |
| map04062 | Chemokine signaling pathway | 89 | 3.09E-20 |
| map04110 | Cell cycle | 80 | 5.45E-30 |
| map04630 | Jak-STAT signaling pathway | 63 | 2.58E-11 |
| map04012 | ErbB signaling pathway | 61 | 5.00E-26 |
| map04350 | TGF-beta signaling pathway | 56 | 2.25E-22 |
| map04210 | Apoptosis | 50 | 2.48E-16 |
| map04115 | p53 signaling pathway | 43 | 2.55E-16 |
| map04370 | VEGF signaling pathway | 43 | 6.08E-14 |
